# Is cortical perfusion a reliable marker for predicting septic acute kidney injury?

**DOI:** 10.1186/s13054-019-2386-9

**Published:** 2019-03-22

**Authors:** Patrick M. Honore, David De Bels, Andrea Gallerani, Rachid Attou, Sebastien Redant, Leonel Barreto Gutierrez, Willem Boer

**Affiliations:** 1 ICU Department, Centre Hospitalier Universitaire Brugmann, 4, Place Arthur Van Gehuchtenplein, 1020 Brussels, Belgium; 2 Adjunct Head of Clinics, Centre Hospitalier Universitaire Brugmann, Brussels, Belgium; 3 Department of Anesthesiology, Intensive Care Medicine, Emergency Medicine and Pain Medicine, Ziekenhuis Oost-Limburg, Genk, Belgium

With interest, we read the recent publication by Harrois et al. correlating low cortical blood flow with the risk of septic acute kidney injury (SAKI) [1]. SAKI is the most common form of AKI, accounting for almost 50% of cases of AKI [2]. The pathophysiology of SAKI is incompletely understood but comprises ischemia-reperfusion damage, direct inflammatory injury, endothelial cell and microcirculatory dysfunction, and apoptotic changes [3]. It has been a long-held belief that a reduced global renal blood flow (RBF) was the driving force responsible for initiating SAKI though this traditional paradigm has been challenged [3]. Harrois, by using renal contrast-enhanced ultrasound (CEUS), observed an average decrease in cortical renal perfusion (CRPF) during septic shock (*N* = 20) compared to patients without (*N* = 10) and an association with SAKI. Closer scrutiny reveals that patients with SAKI trended to higher doses of noradrenaline, lower mean arterial pressure (MAP), and lower cardiac index (CI), without reaching statistical significance. Lactate was significantly higher in SAKI patients.

In a sheep model, Lankadeva demonstrated hemodynamic and oxygenation mismatch between the cortex and medulla, before SAKI became evident. Despite large increases in global RBF and renal oxygen delivery, significant hypoperfusion and hypoxia were detected at the medulla but not at the cortex [4]. This may be due to a sepsis-induced nitric oxide synthase-mediated deficit in intrarenal autoregulatory capacity, rendering the renal medulla more susceptible to hypoxia [4] though the fact that the medulla has no proper vasculature and is dependent on oxygen diffusion may figure [3, 5]. Thus, intrarenal blood redistribution may be a key determinant in initiating SAKI instead of truly reduced RBF even in the cortex [4].

Lankadeva initiated treatment with noradrenaline, restoring MAP to normal and strongly enhancing RBF, however with the effect of further reducing medullary perfusion and oxygenation [4].

Medullar oxygenation, subject to macrocirculatory (with 20% shunting of the renal blood flow) [3] and microcirculatory influences [4, 5] seems relatively independent of the RBF and CRPF [4]. It is our opinion that it is only possible to conclude that averaged reduction in CRPF is truly predictive of SAKI, if renal perfusion is monitored simultaneously, together with (a significant reduction in) medullary oxygenation. With only 20 patients in this cohort, it is fair to ask if patients developing SAKI may simply do so because of an increased burden of sickness and that the findings are an epiphenomenon of disease and therapy [1].

## Abbreviations

AKI: Acute kidney injury
CEUS: Renal contrast-enhanced ultrasound
CI: Cardiac index
CRPF: Cortical renal perfusion
MAP: Mean arterial pressure
RBF: Renal blood flow
SAKI: Septic acute kidney injury
